# Supplementary material for: Antibiotic Resistant Bloodstream Infections in Pediatric Patients Receiving Chemotherapy or Hematopoietic Stem Cell Transplant: Factors Associated with Development of Resistance, Intensive Care Admission and Mortality
Source: Antibiotics (Basel). 2021 Mar 5;10(3):266. doi: 10.3390/antibiotics10030266 (PMC8000765; doi:10.3390/antibiotics10030266)

Supplementary Table S1. Demographic and clinical characteristics of patients at time of 1291 BSI episodes

Total bloodstream infections, n(%)	1291 (100.0)
Male sex	756 (58.6)
Median age at bloodstream infections, years, (interquartile range)	8 (3-13)
Year of bloodstream infections	
2015	426 (33.0)
2016	421 (32.6)
2017	444 (34.4)
Underlying disease	
Hematological malignancy	838 (64.9)
Acute lymphoblastic leukemia	457 (54.5)
Acute myeloid leukemia	232 (27.7)
Non-Hodgkin lymphoma	94 (11.2)
Hemophagocytic lymphohistiocytosis	30 (3.6)
Other leukemias	25 (3.0)
Solid tumor	322 (24.9)
Neuroblastoma	90 (28.0)
Bone and soft tissue sarcoma	78 (24.2)
Central nervous system tumor	70 (21.7)
Hodgkin disease and other solid tumors	84 (26.1)
Non malignant disease receiving HSCT	131 (10.1)
HSCT	320 (24.8)
Autologous	54 (16.9)
Allogeneic	266 (83.1)
Pre-engraftment phase	119 (44.7)
Post-engraftment phase	74 (27.8)
Acute graft vs. host disease (a-GvHD)	44 (16.5)
Chronic graft vs. host disease (c-GvHD)	29 (10.9)
Neutropenia at onset of bloodstream infection	906 (70.2)
Central venous catheter at onset of bloodstream infection	1239 (96.0)
Partially implantable (Hickman-Broviac)	616 (49.7)
Totally implantable (PORT)	283 (22.8)
Peripherally inserted central catheter (PICC)	162 (13.1)
Hickman Broviac + PORT/PICC	8 (0.6)
PICC + PORT	2 (0.2)
Not specified	168 (13.6)
Relapse/progression of underlying malignancy	311 (24.1)
Previous antibacterial exposure (prophylaxis/therapy)	743 (57.6)
Standard regimen active vs *P. aeruginosa*[1]	348 (46.8)
Carbapenems	200 (26.9)
Fluoroquinolones	53 (7.1)
β-lactams not active vs *P. aeruginosa* (β-lactams)	43 (5.8)
Combination[2]	72 (9.7)
Others[3]	27 (3.6)
Previous colonizations with resistant pathogens	231 (17.9)
Previous infections with resistant pathogens	174 (13.5)

[1]Included piperacillin/tazobactam, ceftazidime or cefepime, used alone or with an aminoglycosides.

[2]Combination of two or more of the following fluoroquinolone/ β-lactams not active vs *P. aeruginosa*/ Standard regimen active vs *P. aeruginosa*/ carbapenem.

[3]Others: daptomycin, linezolid, ceftaroline, tigecycline and colistin

Supplementary Table S2. Distribution according to frequency of 1379 (1289 bacteria and 90 fungi) isolated strains in 1291 bloodstream infections in 1031 patients.

	n	%
**Bacteria**	**1289**	**100.0**
Escherichia coli	264	20.5
Staphylococcus aureus	174	13.5
Klebsiella pneumoniae	165	12.8
Viridans streptococci	155	12.0
Pseudomonas aeruginosa	132	10.2
Enterobacter spp	97	7.5
Other non-fermenting Gram-negatives	102	7.9
Stenotrophomonas maltophilia	32	2.5
Bukholderia cepacia	7	0.5
Acinetobacter lwoffii	3	0.2
Acinetobacter pitii	3	0.2
Acinetobacter spp.	3	0.2
Capnocytophaga spp	3	0.2
Moraxella catarrhalis	3	0.2
Ochrobactrum anthropi	3	0.2
Pseudomonas putida	3	0.2
Pseudomonas stutzeri	3	0.2
Achromobacter xylosoxidans	2	0.2
Acinetobacter junii	2	0.2
Moraxella osloensis	2	0.2
Pseudomonas fluorescens	2	0.2
Pseudomonas luteola	2	0.2
Abiotrophia defectiva	1	0.1
Achromobacter denitrificans	1	0.1
Acinetobacter calcoaceticus	1	0.1
Acinetobacter haemolyticus	1	0.1
Acinetobacter ursingii	1	0.1
Bukholderia phenazinium	1	0.1
Capnocytophaga sputigena	1	0.1
Granulicatella adiacens	1	0.1
Moraxella non liquefaciens	1	0.1
Moraxella spp	1	0.1
Ralstonia insidiosa	1	0.1
Ralstonia picketii	1	0.1
Rothia mucilaginosa	1	0.1
Rothia spp.	1	0.1
Sphingobacterium spp	1	0.1
Sphingomonas paucimobilis	1	0.1
Pseudomonas luteola	1	0.1
Not further specified	12	0.9
Enterococcus faecium	81	6.3
Enterococcus faecalis	48	3.7
Other Enterobacteriales	43	3.3
Klebsiella oxytoca	16	1.3
Serratia marcescens	3	0.2

Pantoea agglomerans	3	0.2
Pantoea species	2	0.2
Citrobacter koseri	2	0.2
Enterobacter cloacae	2	0.2
Salmonella enteritidis	2	0.2
Citrobacter brakii	1	0.1
Enterobacter asburiae	1	0.1
Escherichia fergusonii	1	0.1
Leclercia adenocarboxylata	1	0.1
Morganella morganii	1	0.1
Proteus mirabilis	1	0.1
Proteus vulgaris group	1	0.1
Raoultella ornithinolytica	1	0.1
Salmonella sp	1	0.1
Klebsiella ozaenae	1	0.1
Not further specified	3	0.2
Acinetobacter baumannii complex	28	2.2
**Fungi**	**90**	**100.0**
Candida	81	90.0
C.arapsilosis	27	30.0
C.albicans	17	18.9
C.guillermondii	12	13.3
C.krusei	8	8.9
C.tropicalis	5	5.6
C.lusitaniae	3	3.3
C.dubliniensis	2	2.2
C.glabrata	1	1.1
C.kefyr	1	1.1
C.pelliculosa	1	1.1
C.norvegensis	1	1.1
Not further specified	3	3.3
Other yeasts	9	10.0
Saprochaete clavata	3	3.3
Rhodotorula mucilaginosa	2	2.2
Exophiala dermatitidis	1	1.1
Geotricum capitatum	1	1.1
Trichosporon mucoides	1	1.1
Not identified	1	1.1

Supplementary Table S3. Resistance to antibiotics by type of isolated pathogens.

	Susceptible, n	Resistant, n	% resistant, 95% CI	Not tested, n	% Not tested
Escherichia coli, n=264					
Meropenem	230	6	2.5 (1.2-5.3)	28	10.6
Colistin	84	3	3.5 (0.7-15.7)	177	67.0
Amikacin	174	9	4.9 (3.0-8.0)	81	30.7
Gentamycin	211	46	17.9 (12.1-25.7)	7	2.7
Ciprofloxacin	169	78	31.6 (19.9-46.2)	17	6.4
Ceftazidime	149	50	25.1 (12.9-43.1)	65	24.6
Cefepime	132	53	28.6 (13.8-50.2)	79	29.9
Piperacillin tazobactam	163	46	22.0 (15.0-31.1)	55	20.8
Tigecyclin	114	1	0.9 (0.1-6.2)	149	56.4
Ceftolozane tazobactam	1	5	83.3 (5.7-99.7)	258	97.7
Ceftazidime avibactam	1	2	66.7 (0.1-100.0)	261	98.9
Klebsiella pneumoniae, n=165					
Meropenem	122	23	15.9 (8.2-28.3)	20	12.1
Colistin	79	1	1.2 (0.6-2.8)	85	51.5
Amikacin	117	20	14.6 (7.8-25.5)	28	17.0
Gentamycin	100	56	35.9 (18.0-58.8)	9	5.5
Ciprofloxacin	91	71	43.8 (24.0-65.8)	3	1.8
Ceftazidime	47	89	65.4 (38.3-85.3)	29	17.6
Cefepime	52	83	61.5 (26.8-87.4)	30	18.2
Piperacillin tazobactam	75	56	42.7 (24.5-63.2)	34	20.6
Tigecyclin	55	9	14.1 (8.1-23.3)	101	61.2
Ceftazidime avibactam	1	1	50.0 (0.1-100.0)	163	98.8
Enterobacter spp, n=97					
Meropenem	93	0	0	4	4.1
Colistin	39	1	2.5 (0.6-9.2)	57	58.8
Amikacin	72	1	1.4 (0.2-7.3)	24	24.7
Gentamycin	72	23	24.2 (7.9-54.4)	2	2.1

	Susceptible, n	Resistant, n	% resistant, 95% CI	Not tested, n	% Not tested
Ciprofloxacin	84	12	12.5 (3.4-36.3)	1	1.0
Ceftazidime	43	32	42.7 (15.8-74.7)	22	22.7
Cefepime	49	24	32.9 (8.5-72.1)	24	24.7
Piperacillin tazobactam	59	15	20.3 (13.2-29.8)	23	23.7
Tigecyclin	25	1	3.8 (0.5-25.7)	71	73.2
Ceftazidime avibactam	0	1	100	96	99.0
Other Enterobacteriales, n=43					
Meropenem	38	0	0	5	11.6
Colistin	12	1	7.7 (0.3-66.8)	30	69.8
Amikacin	28	0	0	15	34.9
Gentamycin	34	6	15.0 (4.9-37.8)	3	7.0
Ciprofloxacin	36	6	14.3 (5.4-32.7)	1	2.3
Ceftazidime	23	6	20.7 (4.3-60.1)	14	32.6
Cefepime	24	2	7.7 (0.6-53.7)	17	39.5
Piperacillin tazobactam	26	8	23.5 (11.4-42.4)	9	20.9
Tigecyclin	16	1	5.9 (0.4-46.1)	26	60.5
Pseudomonas aeruginosa, n=132					
Meropenem	80	30	27.3 (9.0-58.7)	22	16.7
Colistin	83	0	0	49	37.1
Amikacin	88	16	15.4 (4.2-42.9)	28	21.2
Gentamycin	113	15	11.7 (2.7-38.7)	4	3.0
Ciprofloxacin	106	21	16.5 (3.6-51.3)	5	3.8
Ceftazidime	81	32	28.3 (13.9-49.1)	19	14.4
Cefepime	74	25	25.2 (8.1-56.3)	33	25.0
Piperacillin tazobactam	81	29	26.4 (12.6-47.0)	22	16.7
Ceftolozane tazobactam	1	0	0	131	99.2
Ceftazidime avibactam	1	0	0	131	99.2
Acinetobacter baumannii complex, n=28					
Meropenem	21	7	25.0 (5.4-66.0)	0	0.0
Colistin	12	1	7.7 (0.1-82.2)	15	53.6

	Susceptible, n	Resistant, n	% resistant, 95% CI	Not tested, n	% Not tested
Amikacin	16	7	30.4 (5.2-77.7)	5	17.9
Gentamycin	19	9	32.1 (9.0-69.3)	0	0.0
Ciprofloxacin	20	7	25.9 (5.6-67.4)	1	3.6
Ceftazidime	12	8	40.0 (4.4-90.7)	8	28.6
Cefepime	11	9	45.0 (4.3-93.7)	8	28.6
Piperacillin tazobactam	11	6	35.3 (5.7-83.2)	11	39.3
Tigecyclin	5	2	28.6 (4.5-77.3)	21	75.0
Ceftazidime avibactam	0	1	100	27	96.4
Other *Gram*-negatives, n=102					
Meropenem	34	6	15.0 (0.5-37.2)	62	60.8
Colistin	16	9	36.0 (7.0-80.7)	77	75.5
Amikacin	21	7	25.0 (8.3-55.1)	74	72.5
Gentamycin	27	18	40.0 (18.2-66.6)	57	55.9
Ciprofloxacin	49	9	15.5 (6.9-31.2)	44	43.1
Ceftazidime	36	19	34.5 (23.7-47.2)	47	46.1
Cefepime	13	10	43.5 (22.3-67.3)	79	77.5
Piperacillin tazobactam	19	15	44.1 (21.4-69.6)	68	66.7
Tigecyclin	27	2	6.9 (0.5-52.9)	73	71.6
Ceftolozane tazobactam	1	0	0	101	99.0
Staphylococcus aureus, n=174					
Methicillin	109	22	16.8 (7.9-32.1)	43	24.7
Vancomycin	148	0	0	26	14.9
Daptomycin	72	1	1.4 (0.1-16.2)	101	58.0
Linezolid	138	0	0	36	20.7
Tigecyclin	91	1	1.1 (0.5-2.4)	82	47.1
Viridans streptococci, n=155					
Penicillin	103	40	28.0 (18.7-39.6)	12	7.7
Ampicillin	34	24	41.4 (28.1-56.1)	97	62.6
Enterococcus faecalis, n=48					
Ampicillin	40	5	11.1 (2.6-36.5)	3	6.3

	Susceptible, n	Resistant, n	% resistant, 95% CI	Not tested, n	% Not tested
Vancomycin	43	2	4.4 (0.7-24.4)	3	6.3
Teicoplanin	27	0	0	21	43.8
Daptomycin	9	0	0	39	81.3
Linezolid	27	2	6.9 (1.6-25.3)	19	39.6
Tigecyclin	21	0	0	27	56.3
Enterococcus faecium, n=81					
Ampicillin	7	68	90.7 (80.9-95.7)	6	7.4
Vancomycin	49	32	39.5 (26.3-54.4)	0	0.0
Teicoplanin	28	7	20.0 (6.6-46.8)	46	56.8
Daptomycin	13	3	18.7 (1.5-77.4)	65	80.2
Linezolid	72	1	1.4 (0.1-15.5)	8	9.9
Tigecyclin	61	0	0	20	24.7
Fungal infection-candida, n=81					
Fluconazole	46	17	27.0 (16.2-41.5)	18	22.2

Supplementary Figure S1. Distribution, by centre, of percentages of resistant antibiotic bacteremia among *Gram*-negative BSI

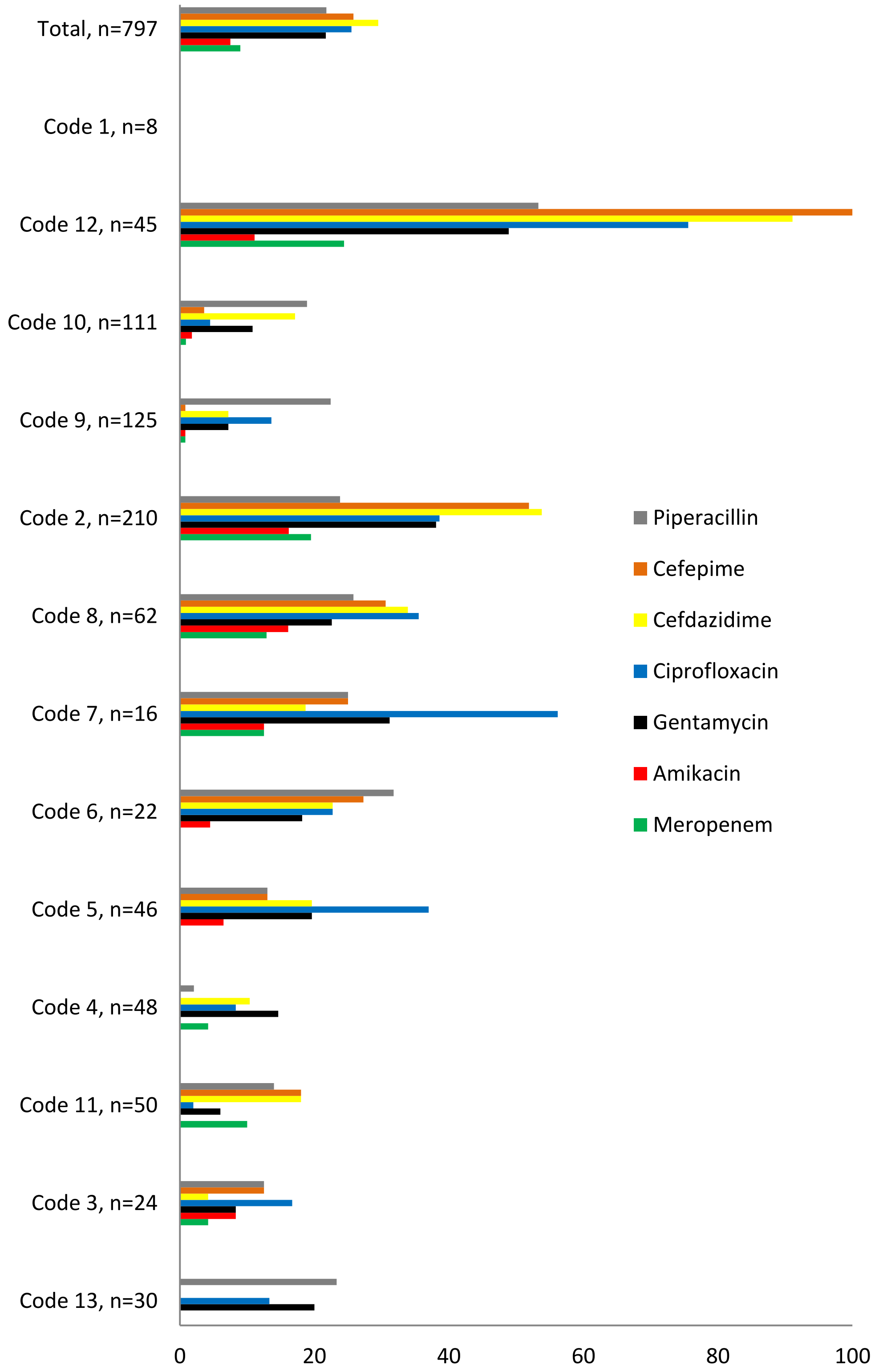

Supplementary Figure S2. Distribution, by centre, of percentages of resistant antibiotic bacteremia among *Gram*-positive BSI

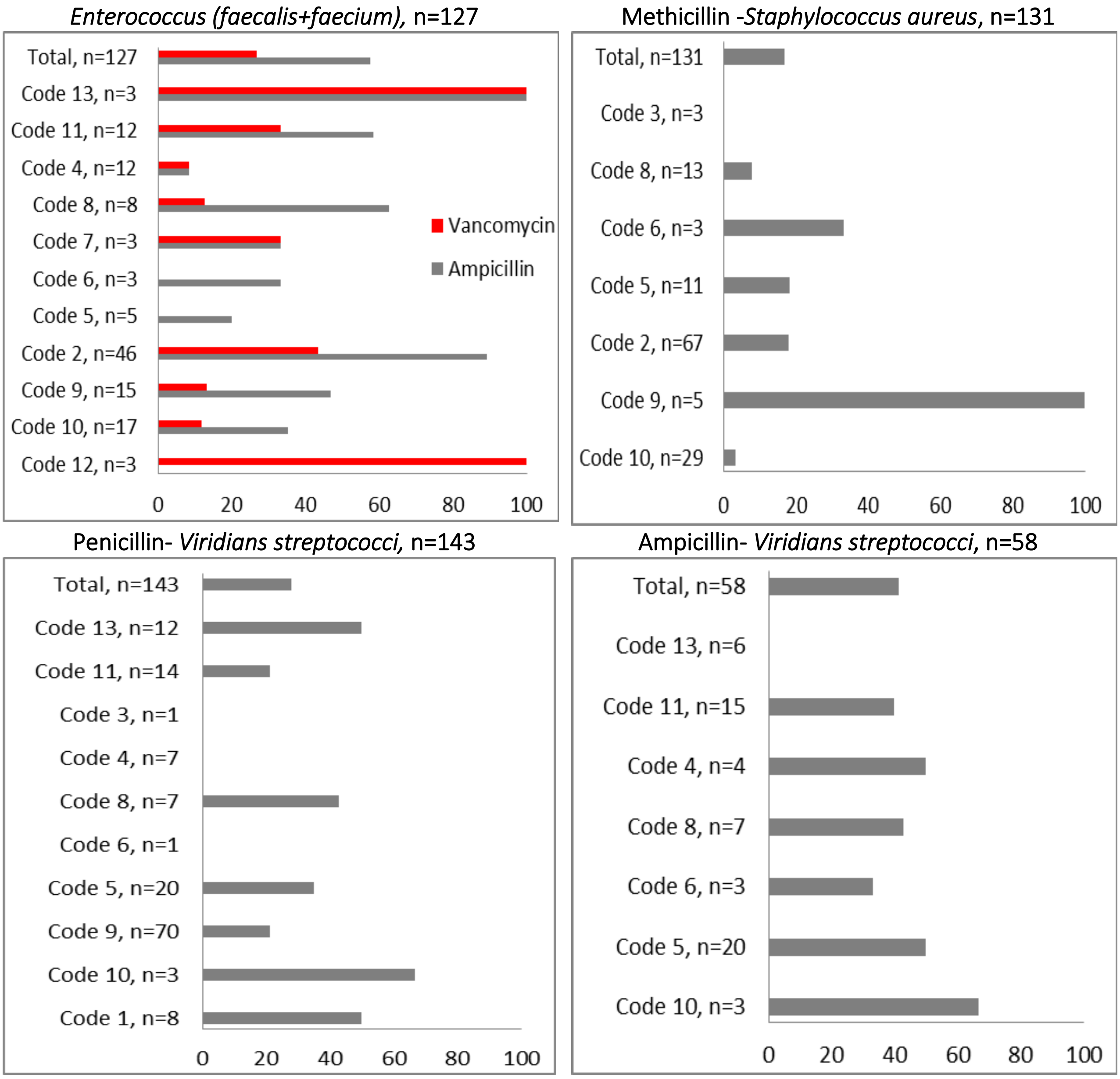

Supplement: Supplementary file 1 [file antibiotics-10-00266-s001.pdf]
